# The use of non-uniform drowning terminology: a follow-up study

**DOI:** 10.1186/s13049-017-0405-x

**Published:** 2017-07-17

**Authors:** Andrew C. Schmidt, Justin R. Sempsrott, David Szpilman, Ana Catarina Queiroga, Matt S. Davison, Ryan J. Zeigler, Sean J. McAlister

**Affiliations:** 1 Department of Emergency Medicine, University of Florida College of Medicine-Jacksonville, 655 W 8th St, 32209 Jacksonville, FL USA; 2 Lifeguards Without Borders, 757 S Iron Springs Ave, 83634 Kuna, ID USA; 3 Sociedade Brasileira de Salvamento Aquático, Av das Américas 3555, Bloco 2, Sala 302, Barra da Tijuca, Rio de Janeiro, RJ 22631-004 Brazil; 4 EPIUnit, Instituto de Saúde Pública, Universidade do Porto, Rua das Taipas, 135, 4050-600 Porto, Portugal; 5 Center for Health Equity and Quality Research, University of Florida College of Medicine-Jacksonville, 655 W 8th St, 32209 Jacksonville, FL USA; 6 Brody School of Medicine, East Carolina University, 600 Moye Blvd, 27834 Greenville, NC USA; 7 Tulane University School of Medicine, Tulane University, 1430 Tulane Ave, 70112 New Orleans, LA USA

## Abstract

**Background:**

In 2002, the World Congress on Drowning developed a uniform definition for drowning. The aim of this study is to determine the prevalence of “non-uniform drowning terminology” (NUDT) and “non-uniform drowning definitions” (NUDD) in peer-reviewed scientific literature from 2010 to 2016, and compare these findings with those from our unpublished study performing a similar analysis on literature from 2003 to 2010.

**Methods:**

A systematic review was performed using drowning-specific search terms in Pubmed and Web of Science. Titles and abstracts published between July 2010 and January 2016 were screened for relevance to the study focus. Articles meeting screening criteria were reviewed for exclusion criteria to produce the final group of studies. These articles were reviewed by four reviewers for NUDT and NUDD. The Fisher exact test was used to determine any statistically significant changes.

**Results:**

The final group of studies included 167 articles. A total of 53 articles (32%) utilized NUDT, with 100% of these including the term “near drowning”. The proportion of articles utilizing NUDT was significantly less than reported by our previous study (*p* < 0.05). In addition, 32% of the articles included a definition for drowning (uniform or non-uniform), with 15% of these utilizing NUDD.

**Discussion:**

Our study reveals a statistically significant improvement over the past thirteen years in the use of uniform drowning terminology in peer-reviewed scientific literature, although year-to-year variability over the current study period does not yield an obvious trend.

**Conclusions:**

Of the articles reviewed during the 2010-2016 study period, 32% included outdated and non-uniform drowning terminology and definitions. While this reveals an absolute decrease of 11% as compared with the previous study period (2003-2010), there is still significant room for improvement.

## Background

Uniform terminology is vital to the effective study of any process in medicine or public health. The study of drowning faces the challenges of addressing prevention, rescue, and treatment across low income and high income settings. Without high quality data, prevention campaigns cannot be targeted towards high risk populations and treatments cannot evolve in concert with new knowledge and technology. When researchers and providers do not speak a common language in regard to a disease or injury, one cannot expect global data to be comparable and, therefore, of high quality. The study and treatment of drowning has long been hampered by a lack of uniform terminology. A 2005 systematic review [1] analyzing peer-reviewed literature from 1966 to 2002 reported 33 different definitions for drowning in 43 articles over that time period. The primary source of variability was in describing whether the patient survived a drowning episode, leading to the common use of the term “near drowning”. In 2002, in an effort to improve surveillance and data collection, the World Congress on Drowning (WCD) developed a uniform definition: “Drowning is the process of experiencing respiratory impairment due to submersion or immersion in a liquid.” [2] This definition includes three possible outcomes: no morbidity, morbidity, or mortality. The uniform definition was subsequently adopted by the World Health Organization, Centers for Disease Control and Prevention, American Heart Association, and European Resuscitation Council. In addition to establishing this definition, the WCD also called for the discontinuation of modifiers such as “near”, “wet”, “dry”, “secondary”, “active”, “passive”, and “silent” to describe drowning.

At the 2011 World Conference on Drowning Prevention in DaNang, Vietnam, two authors of this paper reported the findings of our unpublished systematic review [3], which determined the prevalence of non-uniform drowning terminology (NUDT) and non-uniform drowning definitions (NUDD) in the peer-reviewed scientific literature between January 1, 2003 and July 15, 2010. At that time, 42% of the articles analyzed contained NUDT. The aim of our current study is to evaluate the evolution of NUDT and NUDD use, by performing a similar analysis of the scientific literature published since the end of our last study.

## Methods

We utilized Pubmed and Web of Science to search all peer-reviewed articles from July 16, 2010 to January 1, 2016. The following search terms were used: “drowning”, “drowned”, “submersion”, and “immersion”. These search methods were similar to our original study, except that modifiers (“secondary”, “dry”, “wet”, “active” and “passive”) were not used, as these do not enhance the search. Using RefWorks (ProQuest, Bethesda, MD), the resulting titles and abstracts were screened for inclusion based on relevance to the public health, pathophysiology, prevention, surveillance, and treatment of drowning. To remain consistent with the methods of our original study, case reports were included and purely forensic or microbiologic studies, or non-human experimental studies were excluded. After screening, the titles and abstracts of the initial group of studies were then evaluated for the following exclusion criteria: letters and editorials, inability to obtain full text, inability to obtain an English version, and irrelevance to study focus. From the final group of studies, four of the authors independently evaluated the full text of each article for the presence of NUDT and for any definition to describe drowning. Articles were classified as “NUDT” if they contained non-uniform modifiers such as “near”, “wet”, “dry”, “secondary”, “active”, “passive”, and “silent” to describe drowning. Additionally, articles were classified as “drowning definition present” (DDP) if a specific definition to describe drowning was used, with further sub-classification of NUDD if the definition provided did not closely match the uniform definition developed by the WCD. If NUDT or NUDD were present, but were used in a historical sense or to discuss the issue of incorrect nomenclature, this was not counted against the article. Each reviewer’s findings were tabulated into a spreadsheet and compared for discrepancies. Any discrepancies were discussed by all 4 reviewers and a consensus decision was made as to how to count the finding. Once all pertinent articles were reviewed and properly tabulated, annual incidence and overall prevalence of NUDT and NUDD were determined. When then used the Fisher exact test (2 × 2 table) to compare the two time periods (2003–2010 and 2010–2016) based on NUDT; statistical significance was defined as *P* < 0.05.

## Results

**Fig. 1:**
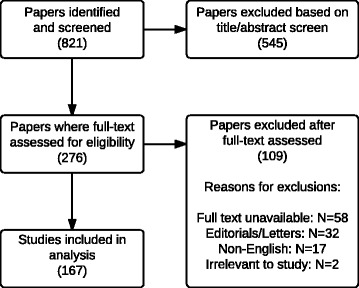
PRISMA diagram of literature search and review

The initial literature search yielded 821 articles. By screening the titles and abstracts, 276 articles were included in the initial group of studies. Of these, 109 met exclusion criteria (32 letters/editorials, 58 unavailable full text, 17 non-English, and 2 irrelevant content) leaving 167 in the final group of studies. The process of the literature search and review is outlined in Fig 1. Reviewing these articles found an overall NUDT prevalence of 32% (53/167). The NUDT present were “near-drowning” (53/53), “secondary drowning” (2/53), and “silent drowning” (1/53). Overall prevalence of DDP for drowning was 32% (54/167), with 15% of this subgroup including NUDD. No articles included both uniform and non-uniform definitions. Details from both study periods can be found in Table 1. Statistical analysis of the prevalence of NUDT over both time periods found the difference to be significant (*P* < 0.05). There was initially a disagreement between evaluators on the presence of NUDT with 15 articles. The most common reason for this was an evaluator missing the existence of NUDT. Another reason for this was confusion over the use of NUDT either in a direct quote from another article or when referencing its historical use. When evaluating for the presence of a drowning definition, initial disagreement occurred with 14 articles. The common reason for this was the presence of unique definitions which did not exactly match that of the WCD, but contained all of the correct elements. All of these instances were easily resolved after careful review of the articles and discussion by the evaluators.

**Table 1:** Annual incidence of non-uniform drowning terminology (NUDT) and non-uniform drowning definitions (NUDD) in peer-reviewed articles, 2010–2016, with comparison to previous study period (2003–2010)

Year	# of Articles	Terminology	Definitions	
		NUDT (%)	DDP	NUDD
2010^a^	15	4 (27)	6	1
2011	31	8 (25)	7	1
2012	22	8 (36)	9	1
2013	25	12 (48)	7	4
2014	29	11 (38)	7	1
2015	45	10 (22)	18	0
TOTAL	167	53 (32)^c^	54	8
2003-2010^b^	227	95 (42)^c^	37	19

*Abbreviatons: NUDT* non-uniform drowning terminology, *DDP* drowning definition present, *NUDD* non-uniform drowning definition

^a^Data period starts on July 16, 2010

^b^Data period ends July 15, 2010, extracted from previous study [3]

^c^Fisher Exact test (2-tailed): *p* = 0.046

## Discussion

In our 2010 analysis of 227 articles published from January 1, 2003 to July 15, 2010, 43% of the articles contained NUDT [3]. Of these, 97% contained the term “near drowning”. Additionally, 16% of the articles contained a definition for drowning; of this sub-group, 52% utilized NUDD. Compared with our original data, our current study reveals an absolute reduction in overall prevalence of NUDT of 11%. In all years except one (2013), the incidence of NUDT was lower than the overall prevalence for 2003–2010, but there was too much year-to-year variability to yield an obvious trend. Similar to our original study, the most common NUDT was “near drowning”, which was present in all articles displaying NUDT. Further analysis found an increased prevalence of a definition for drowning, with the proportion of these articles utilizing NUDD decreasing (37% absolute decrease) from our previous study. Of note, of the articles including a specific definition during the final study year (2015), all of them utilized the uniform definition. These results reveal significant progress towards the utilization of uniform drowning terminology and definitions in the peer-reviewed literature.

As for the reasons behind the continual prevalence of NUDT in the peer-reviewed literature, the authors theorize that this is mainly due to a lack of awareness, on the part of researchers and editors, of the develop of the uniform definition for drowning. To date, no studies have been published evaluating the exact reason or means for improvement, but these should be considered as focuses of future research. Some limitations are apparent with our study. Based on the experience of the authors, there is still confusion on the part of researchers, editors and providers as to which term to use to describe drowning victims who survive the event; it is the opinion of the authors that the term “non-fatal drowning” is most appropriate.

Our study is limited by our initial screening criteria, which excludes articles describing purely forensic or microbiologic studies or non-human experimental studies. While articles within these categories often provide useful data specific to drowning, they were excluded in our initial 2010 study, and these criteria were repeated to best provide comparable data. Additionally, over half of the articles excluded were done so due to our inability to locate full text versions. Often this was due to the abstract only being available within a list of conference presentations.

## Conclusions

Since the development of the uniform definition for drowning in 2002, peer-reviewed scientific articles have continued to include non-uniform drowning terminology. Our study reveals a statistically significant improvement in the use of uniform drowning terminology in the literature, when comparing the 2003–2010 and 2010–2016 time periods, although year-to-year variability over the current study period does not yield an obvious trend.

## Abbreviations

DDP: Drowning definition present
NUDD: Non-uniform drowning definition
NUDT: Non-uniform drowning terminology
WCD: World congress on drowning

## References

[1] Papa L, Hoelle R, Idris A (2005). Systematic review of definitions for drowning incidents. Resuscitation.

[2] Van Beeck EF, Branche CM, Szpilman D (2005). A new definition of drowning: towards documentation and prevention of a global public health problem. Bull World Health Organization.

[3] Sempsrott J, Slattery D, Schmidt A, Penalosa B, Crittle T (2011). Systematic review of non-utstein style drowning terms. Ann Emerg Med.

